# Early use of albumin may increase the risk of sepsis-associated acute kidney injury in sepsis patients: a target trial emulation

**DOI:** 10.1186/s40779-025-00641-z

**Published:** 2025-08-21

**Authors:** Xin-Ya Li, Wei-Sheng Chen, Zhong-Kai Qu, Jian-Guang Chen, Li Li, Shu-Na Li, Yu Wang, Jun Lyu

**Affiliations:** 1 Clinical Research Center, the First Affiliated Hospital of Jinan University, Guangzhou, 510630 China; 2 Department of Intensive Care Unit, the First Affiliated Hospital of Jinan University, Guangzhou, 510630 China; 3 Department of Neurology, the First Affiliated Hospital of Jinan University, Guangzhou, 510630 China; 4 Community Health Service Center of Jinan University, Guangzhou, 510630 China; 5 Department of School Clinic, the First Affiliated Hospital of Jinan University, Guangzhou, 510630 China; 6 Key Laboratory of Regenerative Medicine of the Ministry of Education, Guangzhou, 510630 China

**Keywords:** Target trial emulation, Clone-censor-weight (CCW), Sepsis-associated acute kidney injury (SA-AKI), Albumin

## Abstract

**Background:**

Most sepsis patients develop sepsis-associated acute kidney injury (SA-AKI), which poses a significant threat to survival and lacks specific treatment. To date, there are no published randomized controlled trials that have established a link between albumin use and SA-AKI development in sepsis. Therefore, it is unclear whether albumin use may influence the risk of SA-AKI.

**Methods:**

The present study employed a target trial emulation using observational data to track adult sepsis patients initially admitted to the intensive care unit at Beth Israel Deaconess Medical Center, Boston, Massachusetts, for a period of 7 d from 2008 to 2022. Immortal time bias was controlled using the clone-censor-weight (CCW) method, along with a new-user design to address current user bias. The exposure variable was the early administration of albumin following the onset of sepsis. Based on albumin use, patients were classified into two groups: the albumin group (*n* = 27,088) and the no albumin group (*n* = 27,088). The primary outcome was the development of SA-AKI, and the secondary outcome was 7-day all-cause mortality. The primary outcome was analyzed using competing risk analyses. Furthermore, sensitivity and subgroup analyses were also performed.

**Results:**

Among the 27,088 patients analyzed, albumin administration was associated with a significantly higher SA-AKI risk (relative difference = 3.47%, 95% CI 1.76−5.23) compared to non-administration. There was no clinically meaningful difference in 7-day survival (relative difference = 0.05%, 95% CI −2.30 to 2.45). Sensitivity analyses consistently supported these results. All these analyses were conducted on data that were collected after CCW.

**Conclusions:**

Early albumin administration may increase the risk of SA-AKI in sepsis patients without conferring a short-term survival benefit. These results underscore the need for a rigorous risk-benefit assessment when incorporating albumin into sepsis resuscitation protocols and highlight the need for further clinical validation. However, it is important to exercise caution when interpreting the conclusions of this study, given its exploratory and preliminary nature.

**Supplementary Information:**

The online version contains supplementary material available at 10.1186/s40779-025-00641-z.

## Background

Sepsis-associated acute kidney injury (SA-AKI) is a prevalent and severe complication of sepsis, characterized by a sharp decline in renal function. Its pathogenesis includes systemic inflammatory response, endothelial dysfunction, and microcirculatory disturbances [1–3]. It is a major health concern for critically ill patients, and has been shown to markedly increase in-hospital mortality, as well as contribute to the development of chronic kidney disease and unfavorable long-term outcomes [4–6]. Epidemiological data indicated that 30–80% of patients who suffered from sepsis developed SA-AKI during their stay in the intensive care unit (ICU). This complication posed a serious threat to both survival and quality of life [7]. However, the complex pathogenesis and lack of specific treatments make optimizing therapeutic strategies a major challenge in clinical research [8].

Albumin is a multifunctional protein that has been extensively utilized in the management of critically ill patients. Its function was not limited to colloidal fluid resuscitation, but also included multiple biological properties such as anti-inflammatory, antioxidant, and stabilization of the vascular endothelial barrier [9, 10]. These functions might have been closely linked to the mechanisms underlying SA-AKI onset and progression, potentially protecting renal function by enhancing microcirculatory perfusion, mitigating inflammation, and reducing tissue hypoxia [11, 12]. The 28th Acute Disease Quality Initiative (ADQI) workgroup consensus report posed the research question of whether there was an indication for fluid resuscitation using albumin in SA-AKI patients [7]. However, a review of the extant literature revealed no studies directly examining the association between albumin use and the risk of SA-AKI in sepsis patients.

To explore the potential impact of albumin therapy on the development of SA-AKI in patients suffering from sepsis, the present study will employ a target trial emulation (TTE) approach to simulate an ideal randomized controlled trial (RCT). RCTs are often difficult to implement due to ethical considerations, limited resources, and logistical complexities. By leveraging real-world data, the TTE method enhances the reliability of causal inference by minimizing confounding factors and selection bias through a rigorously defined study objective and treatment strategy [13, 14]. The study will assess the clinical effects of early albumin administration in sepsis patients, providing evidence-based insights to optimize treatment strategies.

## Methods

### Study design and setting

The present study utilized the TTE method based on observational data to assess the impact of early albumin use on the development of SA-AKI in sepsis patients. This study employed the clone-censor-weight (CCW) method to reduce immortal time bias, along with a new-user design to address current user bias [14, 15]. Specifically, to mitigate current user bias, only records of patients receiving albumin for the first time were selected. A detailed explanation of the CCW method is provided in Additional file 1: Methods.

Patients admitted to the ICU between 2008 and 2022 were included in the study, with sepsis diagnosed according to Sepsis-3 criteria, which define sepsis as suspected infection combined with an acute increase in Sequential Organ Failure Assessment (SOFA) score ≥ 2 points and acute kidney injury (AKI) identified using the Kidney Disease Improving Global Outcomes (KDIGO) guidelines [16, 17]. The definition of SA-AKI in this study follows the criteria established by the 28th ADQI workgroup, which defines SA-AKI as AKI occurring within 7 d of sepsis onset [7]. The patient’s survival time was calculated from the baseline to the time of death. The study adhered to the Strengthening the Reporting of Observational Studies in Epidemiology (STROBE) guidelines [18].

### Data source

All data for this study were sourced from the Medical Information Mart for Intensive Care IV (MIMIC-IV), a publicly accessible database that is widely utilized in medical research [19–21]. MIMIC-IV v3.1 comprises hospitalization records from approximately 364,627 patients who received emergency or intensive care treatment at Beth Israel Deaconess Medical Center in Boston from 2008 to 2022 [22]. The database ensures patient anonymity, eliminating the need for informed consent. Access to the data was granted to the authors upon completion of the necessary training and certification [20].

### Eligibility criteria

Patients over the age of 18 who had been diagnosed with sepsis, and who were admitted to the ICU for the first time and had an ICU stay longer than 24 h, participated in this study. The exclusion criteria were as follows: 1) patients who developed AKI before the onset of sepsis, 2) patients who received albumin before sepsis onset, and 3) patients with incomplete or incorrect time records. The exclusion criteria did not include post-baseline information [23].

### TTE

TTE is an approach that constructs a framework, like RCT, from observational data, simulating a design for an ideal trial that could be implemented in practice. By explicitly delineating the temporal configuration of the research question, the inclusion criteria, the intervention strategies, the outcome definitions, and the analytical protocols, TTE seeks to minimize biases such as immortal time bias and prevalent user bias, thereby enhancing the interpretability and the causal validity of the observational findings [13, 14]. Additional file 1: Table S1 provides a comparison of TTE with RCT and observational studies. In this study, following the principles of TTE [13, 14, 23, 24], the study initiation point, exposure assessment window, intervention and control allocation strategies, and outcome monitoring during follow-up were defined with precision. Using the TTE method, we established a causal inference-compliant framework (Additional file 1: Table S2) to better estimate the potential causal impact of early albumin administration on sepsis-related outcomes.

### Treatment strategy and assignment

In this study, the time of sepsis onset was defined as the baseline time. The population was classified into the “Albumin group” (treatment group, *n* = 27,088) and the “No albumin group” (control group, *n* = 27,088) based on whether patients received albumin within 24 h after the onset of sepsis [25]. It is not equivalent to a placebo arm.

### Follow-up and outcomes

Patients were observed until the following outcomes occurred: the onset of SA-AKI, death, discharge, or the expiration of the 7-day follow-up period.

### Covariates

Baseline, time-dependent, and post-baseline covariates were selected for adjustments [24]. The type and codes of each variable are shown in Additional file 1: Table S3. The selection of covariates was primarily informed by literature review [7, 26] and expert clinical knowledge, to identify variables that may plausibly confound the relationship between exposure and outcome. For instance, the SOFA score has been demonstrated to reflect illness severity, which is closely related to liquid management strategies [27]. Comorbidities such as chronic kidney disease (CKD) and diabetes have been demonstrated to modify the outcome risk [7, 26]. A detailed explanation regarding the selection of covariates for inverse probability-censored weighting (IPCW) is provided in Additional file 1: Methods. The final list of adjusted covariates includes age, sex, race, year of admission, type of ICU, weight, length of stay before ICU admission, SOFA score, Acute Physiology Score III (APSIII) score, Charlson Comorbidity Index (CCI), crystalloids dosage, time to antibiotic use, hypertension, diabetes, CKD, artificial colloid use, nephrotoxic drug exposure, vasoactive agent use, mechanical ventilation, and renal replacement therapy (RRT).

### Sensitivity and subgroup analyses

To ensure the robustness of the results, 3 sensitivity analyses were conducted. In the first 2 sensitivity analyses, the follow-up grace periods were adjusted to 12 h and 36 h to assess whether the effect of early albumin use remained consistent across different grace periods. In another sensitivity analysis, patients who had received RRT were excluded, and all subsequent sensitivity analyses were repeated using CCW and the previously described analytical procedures. Finally, subgroup analyses were performed based on age, sex, race, CKD, and septic shock to evaluate the potential influence of these variables on outcomes. All 95% confidence intervals (CIs) for sensitivity and subgroup analyses were obtained using a nonparametric bootstrap method with 1000 repetitions.

### Statistical analysis

This study utilized a TTE framework. In observational data, patient treatment intention is unknown. Therefore, the causal contrasts in the TTE are the per-protocol effect [28]. Continuous variables that did not meet the assumption of a normal distribution were reported as medians with interquartile ranges (IQRs) and compared using the Wilcoxon rank-sum test. Categorical variables were presented as frequencies and percentages, with group differences assessed using the chi-square test.

Initially, each patient in the original dataset was replicated, thus creating 2 identical individuals with identical baseline characteristics. These individuals were then assigned to the treatment and control groups. Following the generation of the cloned dataset, human censoring was applied, meaning that patients who deviated from the planned protocol were censored. Specifically, clones in the treatment group who did not receive albumin within the follow-up grace period and clones in the control group who did receive albumin were censored (Additional file 1: Fig. S1). It is noteworthy that most patients do not receive albumin treatment immediately following the onset of sepsis. Consequently, a follow-up grace period was introduced, and the length of the follow-up grace period was set at 24 h [23, 29]. Finally, IPCW was applied to mitigate selection bias that had been introduced by censoring. The weights were truncated at the 1st and 99th percentiles [30]. Additionally, the mean values of covariates over time were visualized before and after weighting (Additional file 1: Figs. S2, S3).

Subsequent analyses were conducted using CCW datasets. The risk difference was calculated as the relative difference in the incidence of the outcome between the treatment and control groups during the specified follow-up period [28]. The development of weighted cumulative risk curves and weighted Kaplan-Meier survival curves was plotted to illustrate the differences in SA-AKI risk between the treatment and control groups, as well as the differences in 7-day survival. In the SA-AKI analysis, death was considered a competing event. To calculate 95% CIs for the differences in SA-AKI risk, 7-day survival, restricted mean time lost (RMTL) [31], and restricted mean survival time (RMST) [32], a nonparametric bootstrap method with 1000 repetitions was used. However, it should be noted that the use of hazard ratios is not recommended for causal inference [28, 33].

Only variables with a missing rate of 20% or less were included in the final analysis. A comprehensive summary of missingness across all candidate variables is provided in Additional file 1: Fig. S4. For variables with a missing rate below this threshold, random forest (RF) imputation was performed using the “mice” package in R [34]. To ensure robustness, sensitivity analyses were performed using predictive mean matching (PMM) and weighted predictive mean matching (midastouch) (Additional file 1: Fig. S5).

Statistical analyses were performed using R v4.4.2 software (https://www.r-project.org/). Data from the MIMIC-IV databases were extracted using Structured Query Language (SQL) [35], leveraging SQL scripts from the official MIT (Massachusetts Institute of Technology) Laboratory for Computational Physiology GitHub repository (https://github.com/MIT-LCP/mimic-code). *P* < 0.05 (two-tailed) was considered statistically significant.

## Results

### Baseline characteristics

**Fig. 1:**
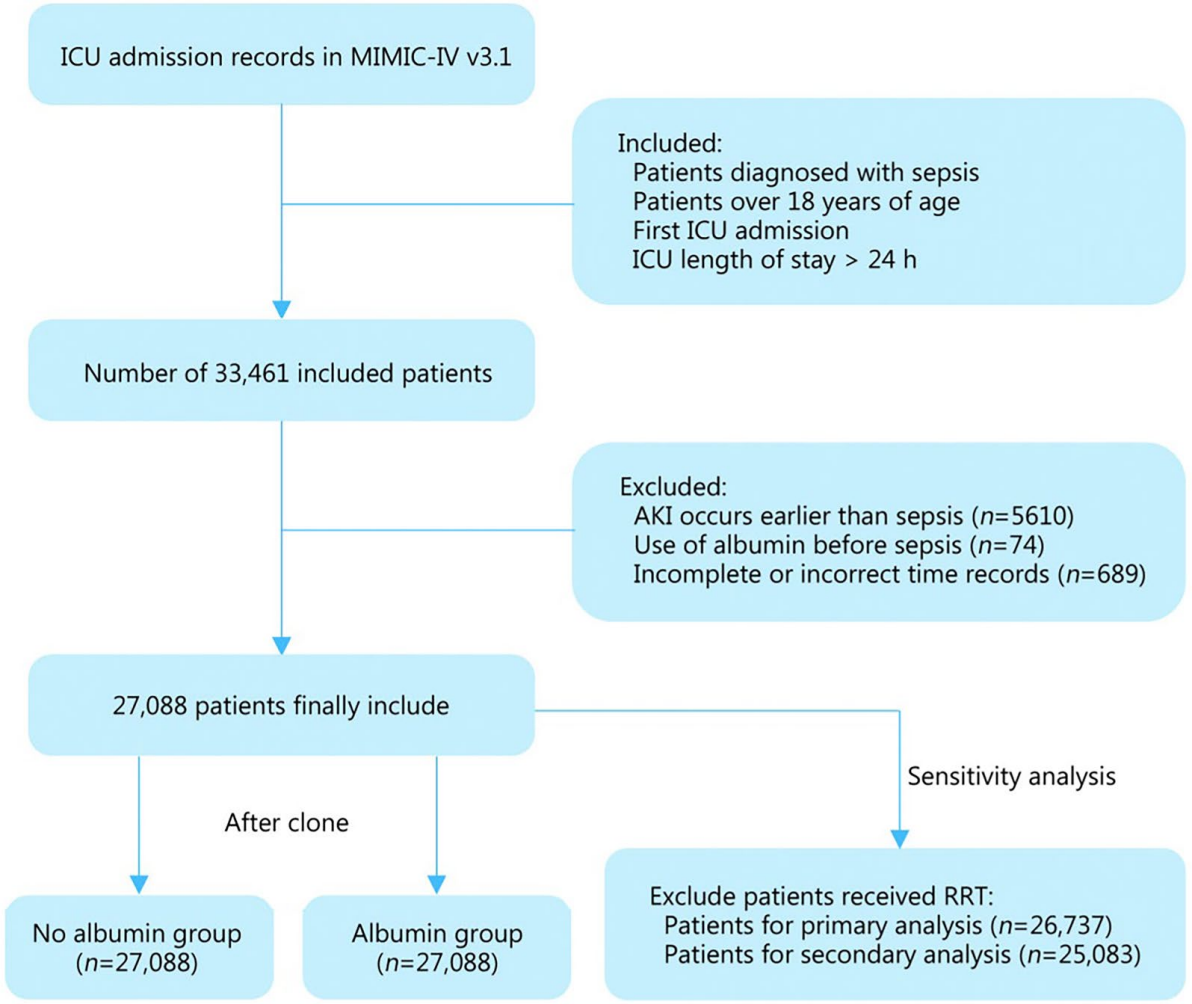
Inclusion and exclusion flowchart of the study. ICU intensive care unit, MIMIC-IV medical information mart for intensive care IV, AKI acute kidney injury, RRT renal replacement therapy

A total of 27,088 patients were included in this study (Fig. 1), with a median age of 68.0 years (IQR 57.0−79.0). Of these patients, 42.4% were female and 57.6% were male. Table 1 provides a detailed summary of baseline characteristics. The median SOFA score of the sepsis patients was 5.0 (IQR 3.0−8.0), and their median length of stay before ICU admission was 1.6 d (IQR 0.6−5.2). The most common comorbidity was hypertension [10,757 (39.7%)], followed by diabetes [8697 (32.1%)] and congestive heart failure [8597 (31.7%)]. Additional file 1: Table S4 presents the baseline characteristics of patients with sepsis in the ICU, excluding those who received RRT in the primary and secondary analyses. These characteristics include demographics, disease severity scores, vital signs, laboratory findings, comorbidities, and treatment details. Additional file 1: Table S5 shows the baseline characteristics of the original data.

**Table 1:** Baseline characteristics of patients with sepsis in the ICU

Variables	Total (*n* = 27,088)
Personal characteristics	
Age [year, median (IQR)]	68.0 (57.0−79.0)
Sex [*n* (%)]	
Male	15,614 (57.6)
Female	11,474 (42.4)
Race [*n* (%)]	
White	18,112 (66.9)
Other*	8976 (33.1)
Type of ICU [*n* (%)]	
CCU	2349 (8.7)
CVICU	5047 (18.6)
MICU	7409 (27.4)
MICU/SICU	5168 (19.1)
Other ICU^#^	7115 (26.3)
Year of admission [*n* (%)]	
2008–2010	9369 (34.6)
2011–2013	6151 (22.7)
2014–2016	5667 (20.9)
2017–2019	3664 (13.5)
2020–2022	2237 (8.3)
LOS before ICU [d, median (IQR)]	1.6 (0.6−5.2)
Weight [kg, median (IQR)]	78.0 (65.3−93.1)
Albumin used in primary analysis [*n* (%)]	
Yes [*n* (%)]	3185 (11.76)
No [*n* (%)]	23,903 (88.24)
Dose [g, median (IQR)]	37.5 (25.0−50.0)
Albumin used in secondary analysis	
Yes [*n* (%)]	5546 (20.47)
No [*n* (%)]	21,542 (79.53)
Dose [g, median (IQR)]	37.5 (25.0−50.0)
Scores on the first day [score, median (IQR)]	
GCS	15.0 (13.0−15.0)
CCI	5.0 (3.0−7.0)
SOFA	5.0 (3.0−8.0)
APSIII	46.0 (35.0−61.0)
Vital signs and laboratory tests on the first day [median (IQR)]	
Temperature (°C)	36.9 (36.6−37.2)
Respiration rate (beat/min)	19.0 (17.0−22.0)
Heart rate (beat/min)	85.0 (75.0−97.0)
Mean blood pressure (mmHg)	75.0 (70.0−82.0)
SpO_2_ (%)	97.3 (95.8−98.5)
White blood cell (10^9^/L)	11.3 (8.0−15.4)
Lymphocyte count (10^9^/L)	1.0 (0.6−1.6)
Neutrophil count (10^9^/L)	9.4 (6.0−13.7)
Monocyte count (10^9^/L)	0.5 (0.3−0.9)
Platelet (10^9^/L)	200.0 (141.0−271.0)
Red cell distribution width (%)	14.6 (13.5−16.2)
Hemoglobin level (g/dl)	10.3 (8.9−11.9)
Creatinine (mg/dl)	1.0 (0.7−1.6)
Bun (mg/dl)	20.0 (14.0−34.0)
Comorbidities [*n* (%)]	
Cerebrovascular disease	
Yes	3879 (14.3)
No	23,209 (85.7)
Myocardial infarct	
Yes	4761 (17.6)
No	22,327 (82.4)
Congestive heart failure	
Yes	8597 (31.7)
No	18,491 (68.3)
Chronic pulmonary disease	
Yes	7445 (27.5)
No	19,643 (72.5)
Renal disease	
Yes	6525 (24.1)
No	20,563 (75.9)
Malignant cancer	
Yes	3570 (13.2)
No	23,518 (86.8)
Liver disease	
Yes	4246 (15.7)
No	22,842 (84.3)
Hypertension	
Yes	10,757 (39.7)
No	16,331 (60.3)
Diabetes	
Yes	8697 (32.1)
No	18,391 (67.9)
Septic shock	
Yes	5608 (20.7)
No	21,480 (79.3)
Chronic kidney disease	
Yes	6395 (23.6)
No	20,693 (76.4)
Drugs and treatments on the first day	
Time to antibiotic use [h, median (IQR)]	7.4 (2.7−16.0)
Total amount of crystalloids dosage [ml, median (IQR)]	2480.0 (1290.8−4000.0)
Artificial colloid [*n* (%)]	
Yes	19 (0.1)
No	27,069 (99.9)
Nephrotoxic drug use^$^ [*n* (%)]	
Yes	9445 (34.9)
No	17,643 (65.1)
Mechanical ventilation ([*n* (%)]	
Yes	12,326 (45.5)
No	14,762 (54.5)
RRT [*n* (%)]	
Yes	351 (1.3)
No	26,737 (98.7)
Vasoactive agent [*n* (%)]	
Yes	11,545 (42.6)
No	15,543 (57.4)
Outcomes [h, median (IQR)]	
Time to SA-AKI occurrence	23.0 (12.0−41.0)
Time to death	74.0 (47.0−141.0)

^*^Other includes individuals identified as Asian, Black, Hispanic/Latino, and Other.

^#^Other ICU includes the following units with smaller sample sizes or mixed clinical profiles: intensive care unit, med/surg intensive care unit, medicine intensive care unit, medicine/cardiology intermediate, neuro intermediate, neuro stepdown, neuro surgical intensive care unit, post anesthesia care unit, surgery/trauma intensive care unit, surgery/vascular/intermediate, and trauma surgical intensive care unit.

^$^The use of nephrotoxic drugs refers to administration from hospital admission until the occurrence of SA-AKI, including vancomycin, gentamicin, amikacin, tobramycin, colistin, polytrim and amphotericin

*ICU* intensive care unit, *CCU* coronary care unit, *CVICU* cardiovascular intensive care unit, *MICU* medical intensive care unit, *SICU* surgical intensive care unit, *LOS* length of stay, *GCS* glasgow coma scale, *CCI* charlson comorbidity index, *SOFA* sequential organ failure assessment, *APSIII* acute physiology score III, *SpO*_*2*_ peripheral capillary oxygen saturation, *BUN* blood urea nitrogen, *RRT* renal replacement therapy

### Primary and secondary outcomes

**Fig. 2:**
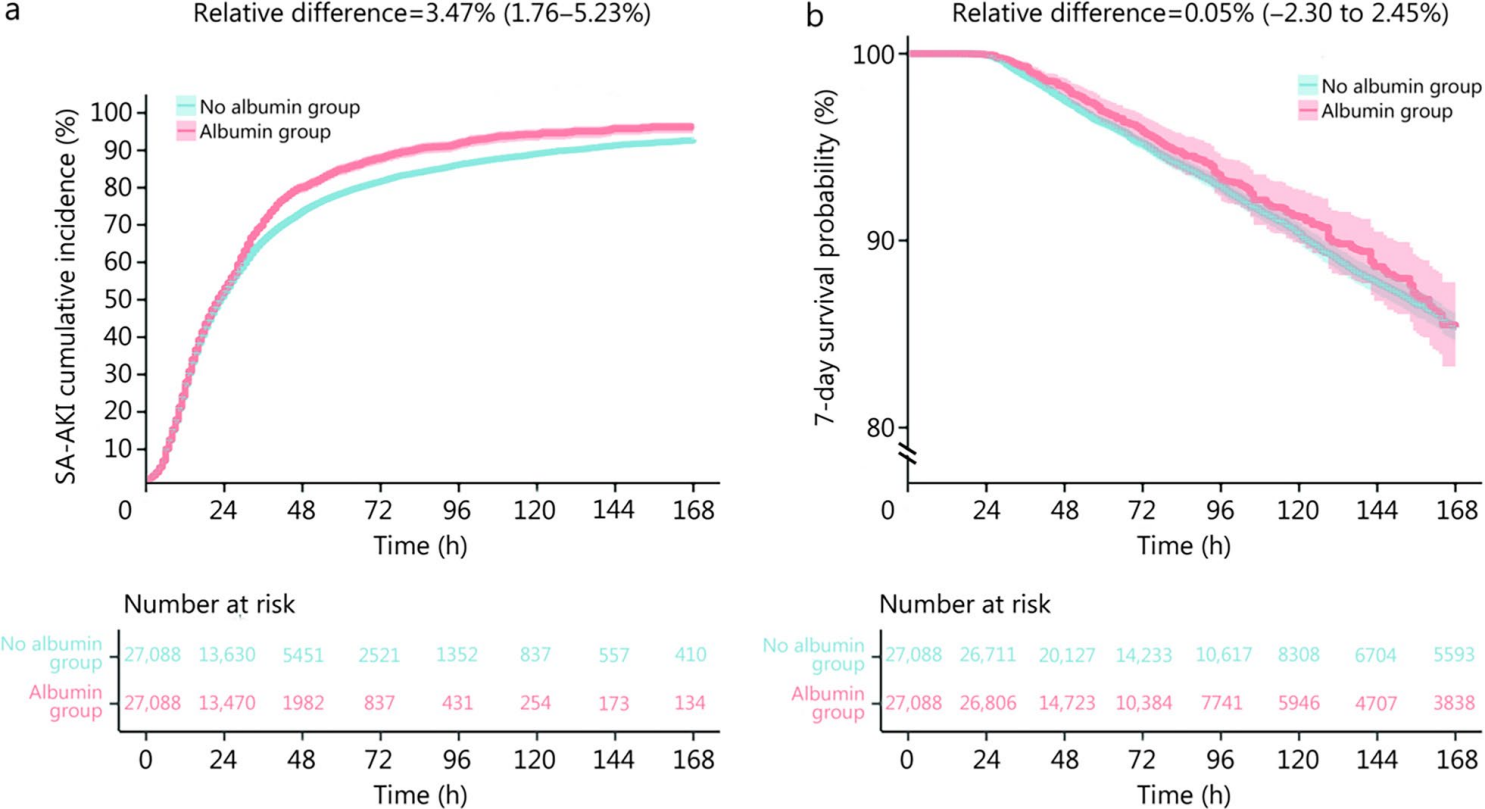
SA-AKI cumulative risk curve and 7-day Kaplan-Meier survival curve after using the clone-censor-weight method. **a** SA-AKI cumulative risk curve. **b** 7-day Kaplan-Meier survival curve. All the 95% CIs were calculated from 1000 bootstrap replicates. SA-AKI sepsis-associated acute kidney injury, CI confidence interval

Utilizing the initiation time of sepsis onset as the starting point for follow-up, the median interval to the onset of SA-AKI was observed as 23.0 h (IQR 12.0−41.0), while the median time to death was determined as 74.0 h (IQR 47.0−141.0) (Table 1). During the 7-day follow-up period, the risk of SA-AKI was 90.71% in the control group and 94.18% in the treatment group (relative difference = 3.41%, 95% CI 1.76−5.23). The 7-day survival rates were 85.40% and 85.35% (relative difference = 0.05%, 95% CI −2.30 to 2.45), respectively (Table 2). The weighted cumulative risk curves for SA-AKI risk between the two groups and the weighted Kaplan-Meier survival curves for 7-day survival are presented in Fig. 2. It is noteworthy that death was treated as a competing event in the SA-AKI analysis, and primary results were derived after adjusting for this competing risk. The risk of SA-AKI was found to be 3.47% (95% CI 1.76−5.23) higher in the treatment group than that in the control group (Fig. 2), indicating an increased risk associated with albumin use. Furthermore, patients using albumin exhibited a prolonged RMTL of 7.69 h (95% CI 5.86−9.52) (Table 2), suggesting SA-AKI may occur earlier in the treatment group, with all observed differences attaining statistical significance. However, the 7-day survival rate in the albumin group was only 0.05% (95% CI −2.30 to 2.45) (Fig. 2**)** higher than in the control group, and the RMST increased by just 0.95 h (95% CI −0.42 to 2.33) (Table 2), indicating that albumin had minimal impact on short-term survival, and with no statistically significant differences.

**Table 2:** Outcomes in patients with sepsis in the ICU after clone-censor-weight

Outcomes (*n* = 54,176)	No albumin group (*n* = 27,088)	Albumin group (*n* = 27,088)
Primary outcome		
SA-AKI (%)	90.71	94.18
Difference in RMTL [h, % (95% CI^*^)]	7.69 (5.86−9.52)	
Difference in SA-AKI risk [% (95% CI^*^)]	3.47 (1.76−5.23)	
Secondary outcome		
7-day survival (%)	85.35	85.40
Difference in RMST [h, % (95% CI^*^)]	0.95 (−0.42 to 2.33)	
Difference in 7-day survival [% (95% CI^*^)]	0.05 (−2.30 to 2.45)	

^*^All 95% CIs were calculated from 1000 bootstrap replicates. *ICU* intensive care unit, *SA-AKI* sepsis-associated acute kidney injury, *RMTL* restricted mean time lost, *CI* confidence interval, *RMST *restricted mean survival time

### Sensitivity and subgroup analyses

Three sensitivity analyses, adjusting for the follow-up grace period, yielded results consistent with the primary analysis (Additional file 1: Tables S6–S7). With a 12 h grace period, the difference in SA-AKI risk between groups was 3.74% (95% CI 1.66−5.92), and the difference in RMTL was 9.40 h (95% CI 7.04−11.78). With a 36 h grace period, the risk difference was 2.85% (95% CI 1.07−4.65), and the RMTL difference was 6.04 h (95% CI 4.38−7.67). Moreover, following the exclusion of patients who had received RRT, the difference in SA-AKI risk remained significant at 3.55% (95% CI 1.62−5.33), with an RMTL difference of 7.79 h (95% CI 5.87−9.56) (Additional file 1: Table S8).

In all sensitivity analyses, the differences in 7-day survival and RMST were minimal and not statistically significant, except for the RMST difference after excluding RRT, which was 1.65 h (95% CI 0.19−3.01). Weighted cumulative risk curves for SA-AKI risk and weighted Kaplan-Meier survival curves for 7-day survival differences between the two groups are presented in Additional file 1: Figs. S6–S8.

**Fig. 3:**
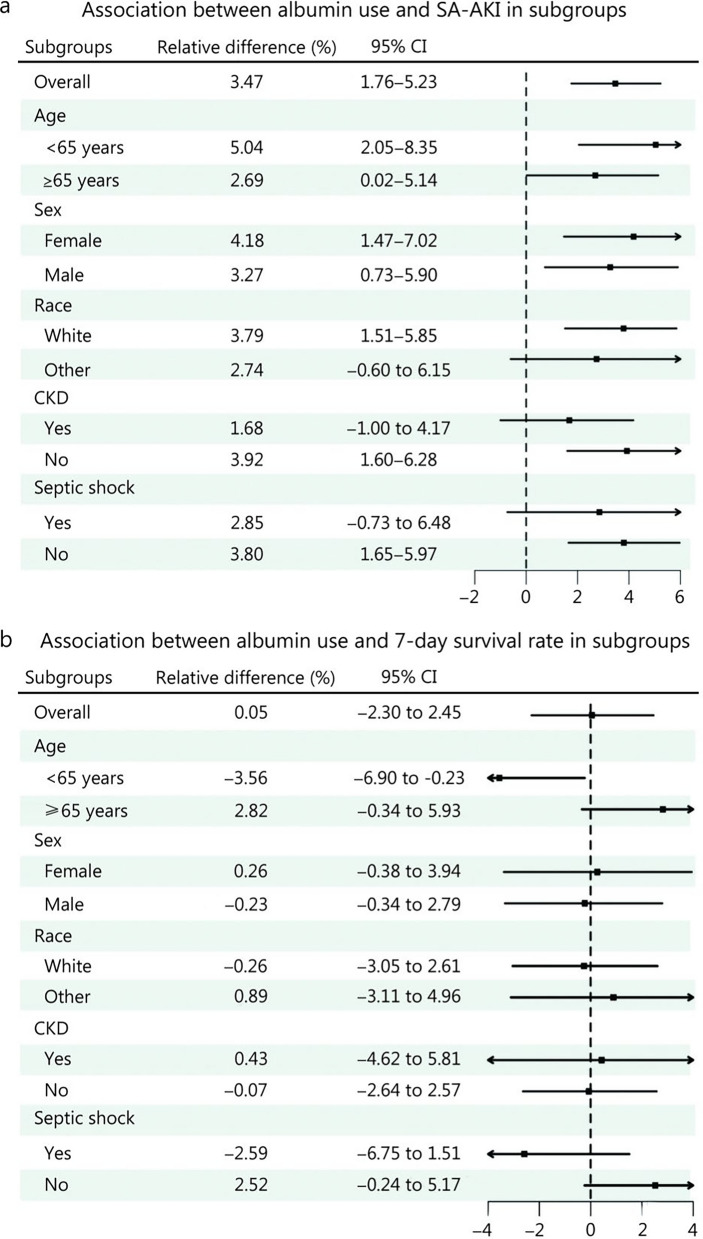
Forest plot for subgroup analysis after using the clone-censor-weight method.** a** Association between albumin use and SA-AKI in subgroups. **b** Association between albumin use and 7-day survival rate in subgroups. All the 95% CIs were calculated from 1000 bootstrap replicates. SA-AKI sepsis-associated acute kidney injury, CI confidence interval, CKD chronic kidney disease

Figure 3 illustrates the association between albumin use and SA-AKI risk as well as 7-day survival across different subgroups. This observation aligns with the results of the main analysis. The risk of SA-AKI differed among the three subgroups of race, CKD, and septic shock. Furthermore, a disparity in 7-day survival was observed exclusively among patients under the age of 65.

## Discussion

This TTE study examined the impact of albumin use on the development of SA-AKI in sepsis patients, adhering to the research design principles of a randomized trial to estimate causal effects from observational data. The findings revealed a significant association between albumin use and an increased risk of SA-AKI, without any observed benefit in short-term mortality. However, the present study is exploratory, and further RCTs are required to confirm these findings.

In patients with sepsis, the development of AKI is influenced by a variety of factors, including systemic inflammation, microcirculatory injury, and direct renal damage. However, it is also impacted by clinical interventions, such as drug administration [3, 36, 37]. Albumin, a pivotal plasma protein necessary for maintaining colloid osmotic pressure and transporting hormones and drugs, is extensively employed in fluid resuscitation for sepsis [9, 38]. However, its effectiveness in preventing SA-AKI or improving prognosis remains debated. The present study aims to provide a preliminary exploration of the association between albumin use and SA-AKI based on the research questions posed in the consensus report of the 28th ADQI workgroup consensus report [7].

When contextualized within existing research, the present findings align with some prior conclusions while also offering new insights into the relationship between albumin use and renal outcomes in sepsis patients. The extant clinical research has not established a significant direct association between albumin use and renal injury in sepsis patients. For instance, the multicenter Saline versus Albumin Fluid Evaluation (SAFE) trial found no significant adverse effects of albumin on renal function in sepsis. The SAFE trial concluded that albumin was comparable to saline for fluid resuscitation, did not cause notable kidney damage, and might have even reduced mortality risk [39]. The potential benefit of albumin can be attributed to its roles in improving hemodynamics, reducing oedema, modulating immune responses, and enhancing microcirculation [40]. However, a number of studies have also found that albumin use does not significantly reduce mortality in patients with sepsis [41, 42], which is consistent with our secondary outcome that albumin does not improve short-term survival. While these studies did not rule out potential adverse effects, the overall evidence suggests that albumin does not directly cause renal injury. In contrast to the findings of existing research [43], our study identified a significant association between early albumin use and an increased risk of SA-AKI. The RMTL results indicated that the treatment group (albumin group) lost 7.69 more hours compared to the control group (no albumin group), meaning that patients in the treatment group developed SA-AKI 7.69 h earlier than those in the control group. However, the direct application of this specific time threshold in clinical practice is not recommended. One potential explanation for this finding is the complexity of fluid load management. Albumin enhances blood volume recovery by improving colloid osmotic pressure. However, excessive fluid administration may instead precipitate or worsen renal injury [44]. This association was also observed in a retrospective cohort study, where albumin use was linked to a higher risk of postoperative AKI complications in major noncardiac surgery [45]. However, no prior studies have compared SA-AKI, as defined by the latest ADQI criteria, about early albumin use. Furthermore, variations in patient populations, indications for albumin administration, and divergent definitions of AKI across studies have compounded the task of conducting direct comparisons with the findings of this study [25, 46, 47].

The utilization of albumin in the management of sepsis remains a subject of controversy, with clinical outcomes exhibiting a high degree of variability due to individual patient factors [48]. The 2021 Surviving Sepsis Campaign (SSC) guidelines advocate for crystalloids as the preferred option for fluid resuscitation in sepsis patients. However, the guidelines also suggest albumin supplementation in adults with sepsis or septic shock requiring large volumes of crystalloids, although this is a weak recommendation with moderate-quality evidence [49]. In contrast, the present study utilized a TTE to demonstrate that albumin use increases the risk of SA-AKI in sepsis patients without impacting 7-day survival. These findings highlight the need for greater caution in clinical albumin use, emphasizing individualized patient assessment, particularly in fluid management. Future research should further refine the optimal strategy for albumin administration in sepsis resuscitation, considering factors such as timing, dosage, and patient-specific conditions to develop a safer, more personalized treatment approach.

## Strengths and limitations

This study has several key strengths. Firstly, the employment of a TTE design effectively minimizes confounding factors, providing more precise causal effect estimates. Secondly, the employment of the MIMIC-IV database, which encompasses a substantial cohort of sepsis patients, serves to enhance the generalizability of the findings. Thirdly, the application of a competing risk model to analyze SA-AKI risk accounts for the competing risk of death, yielding more comprehensive and differentiated conclusions. Furthermore, the robustness of the study is reinforced by the repeated analysis of the data after excluding patients who had undergone RRT and by adjusting for differing follow-up grace periods. This further validates the relationship between albumin use and SA-AKI from some different viewpoints.

The present study has some limitations. First, as with all observational studies, we cannot rule out unmeasured confounders, such as hospital-level variation, or eliminate confounding biases to achieve a perfect balance. Consequently, the necessity for future RCTs to establish causality remains. Secondly, while the data originate from a large medical center, the single-center nature of the study may limit its generalizability to broader patient populations in different regions or healthcare settings. Furthermore, due to the paucity of relevant research for reference, this study did not explore the mechanism of albumin use and increased risk of SA-AKI in depth. Therefore, multicenter studies are required to validate these findings. Thirdly, although this study examined the association between albumin use and SA-AKI, it did not thoroughly investigate other potential kidney protective factors, such as early administration of nephroprotective drugs and hemodynamic management, which could have influenced the results. Moreover, it is important to note that the current approach cannot derive valid interaction *P*-values due to methodological limitations inherent to the CCW framework. These limitations are particularly relevant when considering the complexities arising from data cloning and artificial censoring. Finally, due to limitations in study design and technology, this study did not evaluate the long-term effects of albumin therapy or further analyze the stages of SA-AKI. It is therefore recommended that future research extend follow-up durations to evaluate the sustained impact of albumin therapy on kidney function and overall prognosis in sepsis patients.

## Conclusions

The present study revealed that early albumin administration was significantly associated with a higher risk of SA-AKI, which suggests that albumin may potentiate renal impairment in sepsis patients. These findings emphasize the necessity for a more individualized approach to albumin use in clinical practice, particularly in patients requiring fluid load management or those with preexisting renal dysfunction.

## Supplementary Information

**Additional file 1.** Methods. **Table S1** Comparison of randomized controlled trials, traditional observational studies, and target trial emulation. **Table S2** Specification and emulation of the target trial of the early use of albumin on the occurrence of SA-AKI and 7-day mortality in patients with sepsis. **Table S3** Variable type and codes of the covariates. **Table S4** Baseline characteristics of patients with sepsis in the ICU (excluding those who received RRT in the primary and secondary analysis). **Table S5** Baseline characteristics of original data. **Table S6** Sensitivity analysis: outcomes in patients with sepsis in the ICU after clone-censor-weight (follow-up grace period of 12 h). **Table S7** Sensitivity analysis: outcomes in patients with sepsis in the ICU after clone-censor-weight (follow-up grace period of 36 h). **Table S8 **Sensitivity analysis: outcomes in patients with sepsis in the ICU after clone-censor-weight (excluding those who received RRT in the primary and secondary analysis). **Fig. S1** The clone-censor mechanism in this study. **Fig. S2** Average of changes over time before and after weighting of covariates in the primary analysis. **Fig. S3** Average of changes over time before and after weighting of covariates in the secondary analysis. **Fig. S4** Missing data for all variables in this study. **Fig. S5** Comparison of multiple imputation of candidate features. **Fig. S6** SA-AKI cumulative risk curve and 7-day Kaplan-Meier survival curve (follow-up grace period of 12 h). **Fig. S7** SA-AKI cumulative risk curve and 7-day Kaplan-Meier survival curve (follow-up grace period of 36 h). **Fig. S8** SA-AKI cumulative risk curve and 7-day Kaplan-Meier survival curve (excluding those who received RRT).

## Data Availability

The data were available on the MIMIC-IV website at https://mimic.physionet.org/. The data in this article can be reasonably applied to the corresponding author.

## Abbreviations

ADQI: Acute disease quality initiative
AKI: Acute kidney injury
CCW: Clone-censor-weight
CI: Confidence interval
CKD: Chronic kidney disease
ICU: Intensive care unit
IPCW: Inverse probability-censored weighting
IQR: Interquartile range
KDIGO: Kidney disease improving global outcomes
MIMIC-IV: Medical information mart for intensive care IV
MIT: Massachusetts Institute of Technology
RMST: Restricted mean survival time
RMTL: Restricted mean time lost
RRT: Renal replacement therapy
SA-AKI: Sepsis-associated acute kidney injury
SAFE: Saline versus albumin fluid evaluation
SOFA: Sequential organ failure assessment
SQL: Structured query language
SSC: Surviving sepsis campaign
STROBE: Strengthening the reporting of observational studies in epidemiology
TTE: Target trial emulation
