# Supplementary material for: Early use of albumin may increase the risk of sepsis-associated acute kidney injury in sepsis patients: a target trial emulation
Source: Mil Med Res. 2025 Aug 21;12:51. doi: 10.1186/s40779-025-00641-z (PMC12369241; doi:10.1186/s40779-025-00641-z)
Supplement: Supplementary file 1 — Additional file 1. Methods. Table S1 Comparison of randomized controlled trials, traditional observational studies, and target trial emulation. Table S2 Specification and emulation of the target trial of the early use of albumin on the occurrence of SA-AKI and 7-day mortality in patients with sepsis. Table S3 Variable type and codes of the covariates. Table S4 Baseline characteristics of patients with sepsis in the ICU (excluding those who received RRT in the primary and secondary analysis). Table S5 Baseline characteristics of original data. Table S6 Sensitivity analysis: outcomes in patients with sepsis in the ICU after clone-censor-weight (follow-up grace period of 12 h). Table S7 Sensitivity analysis: outcomes in patients with sepsis in the ICU after clone-censor-weight (follow-up grace period of 36 h). Table S8 Sensitivity analysis: outcomes in patients with sepsis in the ICU after clone-censor-weight (excluding those who received RRT in the primary and secondary analysis). Fig. S1 The clone-censor mechanism in this study. Fig. S2 Average of changes over time before and after weighting of covariates in the primary analysis. Fig. S3 Average of changes over time before and after weighting of covariates in the secondary analysis. Fig. S4 Missing data for all variables in this study. Fig. S5 Comparison of multiple imputation of candidate features. Fig. S6 SA-AKI cumulative risk curve and 7-day Kaplan-Meier survival curve (follow-up grace period of 12 h). Fig. S7 SA-AKI cumulative risk curve and 7-day Kaplan-Meier survival curve (follow-up grace period of 36 h). Fig. S8 SA-AKI cumulative risk curve and 7-day Kaplan-Meier survival curve (excluding those who received RRT). [file 40779_2025_641_MOESM1_ESM.pdf]

Methods

Detailed description of the clone-censor-weight (CCW) method in this study

Clone

In the initial phase of the study, all patients who met the inclusion and exclusion criteria were cloned, to simulate randomization and mitigate baseline confounding bias [1]. The baseline information of each patient was duplicated, thus generating two identical samples, which were subsequently assigned to the treatment and control groups. This cloning process resulted in a dataset that was twice the size of the original. Consequently, the treatment and control groups shared identical baseline covariates, ensuring comparability since each sample was replicated directly from the original data.

Censor

Following data cloning, artificial censoring was applied to exclude samples that deviated from the intended protocol [2]. In this study, clones assigned to the albumin group were censored if albumin was not administered within the designated follow-up grace period (e.g., Patient 2 and Patient 4 in **Additional file 1: Fig. S1**). Conversely, clones assigned to no albumin group were censored if they received albumin during the follow-up grace period (e.g., Patient 1 and Patient 3 in **Additional file 1: Fig. S1**). During the grace period, if a patient is lost to follow-up (which may include hospitalization or ICU discharge) or has an outcome, the patient is treated differently depending on albumin use (e.g. Patient 5, Patient 6, and Patient 7 in **Additional file 1: Fig. S1**).

Weight

The artificial deletion of cloned data is known to introduce selection bias, given the inherent randomness of the process. To address this issue, the inverse probability-censored weighting (IPCW) method was employed [3]. This method aims to reduce bias and yield unbiased estimates by assigning appropriate weights to uncensored data, thereby compensating for the absence of censored data. Specifically, a logistic regression model was fitted at each time point (hourly) to estimate the probability of remaining uncensored. The response variable in the model was a binary indicator of censoring status (1 = censored; 0 = uncensored), and the independent variables included a set of baseline and time-varying covariates and post-baseline covariates. To prevent extreme weights from distorting the estimates, truncation at the 1st and 99th percentiles was applied, enhancing both variance

control and robustness [4,5]. Subsequently, a non-parametric bootstrap method with 1000 replications was utilized to calculate the 95% confidence intervals for differences in restricted mean time lost (RMTL), restricted mean survival time (RMST), incidence, and survival, effectively addressing the variance distortion caused by the cloning process.

This 3-step approach, involving CCW, has been shown to effectively mitigate immortal time bias in target trial emulation and enable the estimation of absolute risk while accounting for time-varying confounders [6].

Detailed description of covariate selection with inverse probability censored weighting (IPCW)

The covariates adjusted for in IPCW can be categorized into three groups [1]: baseline covariates, time-varying covariates, and post-baseline covariates.

Baseline covariates

These are variables available at the study's outset that may influence both treatment selection (i.e., human censoring) and the outcome of interest.

Time-varying covariates

These factors fluctuate over the study period and may impact treatment decisions and outcomes.

Post-baseline covariates

These variables emerge after baseline, potentially affecting treatment uptake and outcomes, necessitating adjustment to mitigate selection bias.

Table S1 Comparison of randomized controlled trials (RCT), traditional observational studies, and target trial emulation (TTE)

Dimension	RCT	Traditional observational study	TTE
Study objective	Evaluate causal effects under controlled conditions	Assess associations or infer potential causality	Emulate a hypothetical RCT to estimate causal effects
Intervention allocation	Randomized assignment	Naturally occurring or clinician-directed	Emulated randomization based on pre-defined strategies
Time zero	Explicitly defined (at randomization)	Often unclear; susceptible to immortal time bias	Explicitly defined by the RCT
Eligibility criteria	Strictly controlled to ensure population homogeneity	Often broad; constrained by data availability	Closely aligned with the RCT, applied to observational data
Data source	Prospectively collected trial data	Retrospective or prospective real-world data	Retrospective observational data (e.g., EHRs, registries, cohorts)
Main advantage	High internal validity; strongest basis for causal inference	Resource-efficient, widely applicable	Enables causal inference when RCT is infeasible or unethical
Bias control	Confounding is minimized through randomization	Susceptible to confounding, selection bias, and measurement bias	Bias minimized via explicit design, careful adjustment, and emulation of trial conditions
Analytical approach	Intention-to-treat (ITT) or per-protocol (PP) analysis	Regression models, propensity scores, stratification, etc.	Emulated PP analysis using causal inference methods (e.g., IPCW, CCW)
Causal inference strength	High, gold standard for causal inference	Low to moderate, depends on assumptions	Moderate to high, contingent on design validity and appropriate adjustment
Ethical and logistical constraints	High cost and ethical restrictions may limit feasibility	Lower cost and typically exempt from interventional ethics	Moderate; uses existing data but requires rigorous methodological planning

EHR electronic health record, *IPCW* inverse probability-censored weighting, *CCW* clone-censor-weight

Table S2 Specification and emulation of the target trial of the early use of albumin on the occurrence of SA-AKI and 7-day mortality in patients with sepsis

Protocol component	Target trial	Emulation in the MIMIC database
Aim	To evaluate whether early albumin use may influence the risk of SA-AKI and short-term mortality	Same as that of the target trial
Eligibility criteria	This study includes sepsis patients aged 18 years or older who are first admitted to the ICU and have an ICU stay of more than 24 h. Patients who had an AKI before the baseline or had already used albumin were excluded from the study. In addition, patients with incomplete or incorrect time records were excluded	Same as that of the target trial, but we did not include patients who had received RRT in one of our sensitivity analyses
Treatment strategies	Use albumin within 24 h of baseline;<br>No albumin use within 24 h of baseline	Same as that of the target trial, but in the context of emulating the target trial using big data, a designated follow-up grace period is permitted [7]. Furthermore, this study implemented an adjustment to the follow-up grace period during sensitivity analyses
Assignment procedures	Patients were randomly assigned to either of two strategies at baseline	Same as that of the target trial, but we used cloning to achieve randomization
Follow-up period	The follow-up period began at the baseline and ended with a diagnosis of SA-AKI, death, loss to follow-up, or 7-day follow-up, whichever occurred first	Same as that of the target trial, but when the cloned data did not match the assigned policy, the clone was censored. The introduced bias was corrected by inverse probability censoring weighting
Primary and secondary outcomes	SA-AKI occurrence (primary outcome) and 7-day all-cause mortality (secondary outcome) in patients with sepsis during the 7-day follow-up period	Same as that of the target trial
Causal contrasts	Intention-to-treat effect;<br>per-protocol effect	We cannot know from retrospective data what the patients' intention to treat was, so only the per-protocol effect applies to big data emulation target trials
Analysis plan	Differences and 95% CIs for 7-day survival/SA-AKI risk, RMTL, and RMST between the two groups were analyzed	Same as that of the target trial, but the data were cloned-censored-weighted before analysis to remove the eternal time bias.

SA-AKI sepsis-associated acute kidney injury, *AKI* acute kidney injury, *MIMIC* medical information mart for intensive care, *ICU* intensive care unit, *RRT* renal replacement therapy, *RMTL* restricted mean time lost, *RMST* restricted mean survival time, *CI* confidence interval

Table S3 Variable type and codes of the covariates

Covariates	Variable type	Codes for categorical variables
Age	Continuous variable	
Sex	Binary variable	1 = Male; 0 = Female
Race	Binary variable	1 = White; 0 = Other*
Year of admission	Polytomous variable	1 = 2008 – 2010; 2 = 2011 – 2013; 3 = 2014 – 2016; 4 = 2017 – 2019; 5 = 2020 – 2022
Type of ICU	Polytomous variable	1= CCU; 2 = CVICU; 3 = MICU; 4 = MICU/SICU; 5 = Other ICU#
Weight	Continuous variable	
LOS before ICU	Continuous variable	
SOFA	Continuous variable	
APSIII	Continuous variable	
CCI	Continuous variable	
Crystalloids dosage	Continuous variable	
Time to antibiotic use	Continuous variable	
Hypertension	Binary variable	1 = Yes; 0 = No
Diabetes	Binary variable	1 = Yes; 0 = No
Chronic kidney disease	Binary variable	1 = Yes; 0 = No
Artificial colloid	Binary variable	1 = Yes; 0 = No
Nephrotoxic drug$	Binary variable	1 = Yes; 0 = No
Vasoactive agent	Binary variable	1 = Yes; 0 = No
Mechanical ventilation	Binary variable	1 = Yes; 0 = No
RRT	Binary variable	1 = Yes; 0 = No

*Other includes individuals identified as Asian, Black, Hispanic/Latino, and Other. #Other ICU includes the following units with smaller sample sizes or mixed clinical profiles: intensive care unit, med/surg intensive care unit, medicine intensive care unit, medicine/cardiology intermediate, neuro intermediate, neuro stepdown, neuro surgical intensive care unit, post anesthesia care unit, surgery/trauma intensive care unit, surgery/vascular/intermediate, and trauma surgical intensive care unit. $The use of nephrotoxic drugs refers to administration from hospital admission until the occurrence of SA-AKI, including vancomycin, gentamicin, amikacin, tobramycin, colistin, polytrim, and amphotericin [8]. SOFA, APSIII, CCI, RRT, mechanical ventilation, crystalloids dosage, time to antibiotic, artificial colloid, vasoactive agent, and weight were all the first records within 24 h of the patient's admission to the ICU. *CCU* coronary care unit, *CVICU* Cardiovascular intensive care unit, *MICU* medical intensive care unit, *SICU* surgical intensive care unit, *LOS* length of stay, *ICU* intensive care unit, *SOFA* sequential organ failure assessment, *APSIII* acute physiology score III, *CCI* charlson comorbidity index, *RRT* renal replacement therapy

Table S4 Baseline characteristics of patients with sepsis in the ICU (excluding those who received RRT in the primary and secondary analysis)

**Variables**	**Patients for primary analysis (*n* = 26,737)**	**Patients for secondary analysis (*n* = 25,083)**
**Personal characteristics**		
Age [year, median (IQR)]	68.0 (57.0 – 79.0)	68.0 (57.0 – 79.0)
Sex [*n* (%)]		
Male	15,416 (57.7)	14,415 (57.5)
Female	11,321 (42.3)	10,668 (42.5)
Race [*n* (%)]		
White	17,952 (67.1)	17,025 (67.9)
Other*	8785 (32.9)	8058 (32.1)
Type of ICU [*n* (%)]		
CCU	2311 (8.6)	2132 (8.5)
CVICU	5019 (18.8)	4826 (19.2)
MICU	7237 (27.1)	6498 (25.9)
MICU/SICU	5116 (19.1)	4881 (19.5)
Other ICU#	7054 (26.4)	6746 (26.9)
Year of admission [*n* (%)]		
2008 – 2010	9204 (34.4)	8609 (34.3)
2011 – 2013	6084 (22.8)	5795 (23.1)
2014 – 2016	5621 (21.0)	5324 (21.2)
2017 – 2019	3621 (13.5)	3343 (13.3)
2020 – 2022	2207 (8.3)	2012 (8.0)
LOS before ICU [d, median (IQR)]	1.6 (0.6 – 5.3)	1.7 (0.7 – 5.7)
Weight [kg, median (IQR)]	78.0 (65.3 – 93.2)	78.0 (65.1 – 92.9)
Albumin used [*n* (%)]		
Yes	3162 (11.83)	5065 (20.19)
No	23,575 (88.17)	20,018 (79.81)
**Scores on the first day [score, median (IQR)]**		
GCS	15.0 (13.0 – 15.0)	15.0 (13.0 – 15.0)
CCI	5.0 (3.0 – 7.0)	5.0 (3.0 – 7.0)
SOFA	5.0 (3.0 – 8.0)	5.0 (3.0 – 7.0)
APSIII	46.0 (34.0 – 61.0)	45.0 (34.0 – 59.0)
**Vital signs and laboratory tests on the first day [median (IQR)]**		
Temperature (°C)	36.9 (36.6 – 37.2)	36.9 (36.6 – 37.2)
Respiration rate (beat/min)	19.0 (17.0 – 22.0)	19.0 (17.0 – 22.0)
Heart rate (beat/minute)	85.0 (75.0 – 97.0)	85.0 (75.0 – 97.0)
Mean blood pressure (mmHg)	75.0 (70.0 – 82.0)	76.0 (70.0 – 82.0)
SpO_2 (%)	97.3 (95.8 – 98.5)	97.3 (95.8 – 98.5)
White blood cell (10^9/L)	11.3 (8.1 – 15.3)	11.3 (8.1 – 15.2)
Lymphocyte count (10^9/L)	1.0 (0.6 – 1.6)	1.1 (0.6 – 1.6)
Neutrophil count (10^9/L)	9.4 (6.0 – 13.6)	9.3 (6.0 – 13.5)
Monocyte count (10^9/L)	0.5 (0.3 – 0.9)	0.5 (0.3 – 0.9)

**Variables**	**Patients for primary analysis (*n* = 26,737)**	**Patients for secondary analysis (*n* = 25,083)**
Platelet (10^9/L)	200.0 (141.0 − 271.0)	201.0 (142.0, 272.0)
Red cell distribution width (%)	14.5 (13.4 − 16.1)	14.5 (13.4 − 16.0)
Hemoglobin level (g/dl)	10.3 (8.9 − 11.9)	10.4 (8.9 − 11.9)
Creatinine (mg/dl)	1.0 (0.7 − 1.5)	1.0 (0.7 − 1.4)
BUN (mg/dl)	20.0 (14.0 − 34.0)	20.0 (14.0 − 32.0)
**Comorbidities [*n* (%)]**		
Cerebrovascular disease		
Yes	3841 (14.4)	3623 (14.4)
No	22,896 (85.6)	21,460 (85.6)
Myocardial infarct		
Yes	4671 (17.5)	4280 (17.1)
No	22,066 (82.5)	20,803 (82.9)
Congestive heart failure		
Yes	8418 (31.5)	7653 (30.5)
No	18,319 (68.5)	17,430 (69.5)
Chronic pulmonary disease		
Yes	7346 (27.5)	6899 (27.5)
No	19,391 (72.5)	18,184 (72.5)
Renal disease		
Yes	6246 (23.4)	5207 (20.8)
No	20,491 (76.6)	19,876 (79.2)
Malignant cancer		
Yes	3545 (13.3)	3402 (13.6)
No	23,192 (86.7)	21,681 (86.4)
Liver disease		
Yes	4175 (15.6)	3678 (14.7)
No	22,562 (84.4)	21,405 (85.3)
Hypertension		
Yes	10,723 (40.1)	10,458 (41.7)
No	16,014 (59.9)	14,625 (58.3)
Diabetes		
Yes	8497 (31.8)	7739 (30.9)
No	18,240 (68.2)	17,344 (69.1)
Septic shock		
Yes	5513 (20.6)	4759 (19.0)
No	21,224 (79.4)	20,324 (81.0)
Chronic kidney disease		
Yes	6117 (22.9)	5090 (20.3)
No	20,620 (77.1)	19,993 (79.7)
**Drugs and treatments on the first day**		
Time to antibiotic use [h, median (IQR)]	7.4 (2.7, 16.1)	7.4 (2.6, 16.2)
Total amount of crystalloids dosage [ml, median (IQR)]	2489.0 (1300.0 − 3996.0)	2491.0 (1316.0 − 3946.0)

Variables	Patients for primary analysis (*n* = 26,737)	Patients for secondary analysis (*n* = 25,083)
Artificial colloid [*n* (%)]		
Yes	19 (0.1)	29 (0.1)
No	26,718 (99.9)	25,054 (99.9)
Nephrotoxic drug use$ [*n* (%)]		
Yes	9280 (34.7)	8634 (34.4)
No	17,457 (65.3)	16,449 (65.6)
Mechanical ventilation [*n* (%)]		
Yes	12,200 (45.6)	11,315 (45.1)
No	14,537 (54.4)	13,768 (54.9)
Vasoactive agent [*n* (%)]		
Yes	11,385 (42.6)	10,430 (41.6)
No	15,352 (57.4)	14,653 (58.4)
**Outcomes [h, median (IQR)]**		
Time to SA-AKI occurrence	23.0 (12.0 – 41.0)	25.0 (13.0 – 43.0)
Time to death	74.0 (47.0 – 141.0)	72.0 (46.0 – 131.0)

*Other includes individuals identified as Asian, Black, Hispanic/Latino, and Other. #Other ICU includes the following units with smaller sample sizes or mixed clinical profiles: intensive care unit, med/surg intensive care unit, medicine intensive care unit, medicine/cardiology intermediate, neuro intermediate, neuro stepdown, neuro surgical intensive care unit, post anesthesia care unit, surgery/trauma intensive care unit, surgery/vascular/intermediate, and trauma surgical intensive care unit. $The use of nephrotoxic drugs refers to administration from hospital admission until the occurrence of SA-AKI, including vancomycin, gentamicin, amikacin, tobramycin, colistin, polytrim and amphotericin [8]. *BUN* blood urea nitrogen, *ICU* intensive care unit, *CCU* coronary care unit, *CVICU* cardiovascular intensive care unit, *MICU* medical intensive care unit, *SICU* surgical intensive care unit, *LOS* length of stay, *GCS* glasgow coma scale, *CCI* charlson comorbidity index, *SOFA* sequential organ failure assessment, *APSIII* acute physiology *Score III*, SpO_2 peripheral capillary oxygen saturation

Table S5 Baseline characteristics of original data

Variables	Total (n = 27,088)	No albumin (n = 21,303)	Albumin (n =5785)	P-value
**Personal characteristic**				
Age [year, median (IQR)]	68.0 (57.0, 79.0)	68.0 (57.0, 79.0)	67.0 (57.0, 76.0)	< 0.001
Sex [n (%)]				< 0.001
Male	15,614 (57.6)	12,086 (56.7)	3528 (61.0)	
Female	11,474 (42.4)	9217 (43.3)	2257 (39.0)	
Race [n (%)]				< 0.001
White	18,112 (66.9)	14,022 (65.8)	4090 (70.7)	
Other*	8976 (33.1)	7281 (34.2)	1695 (29.3)	
Type of ICU [n (%)]				< 0.001
CCU	2349 (8.7)	2272 (10.7)	77 (1.3)	
CVICU	5047 (18.6)	2154 (10.1)	2893 (50.0)	
MICU	7409 (27.4)	6496 (30.5)	913 (15.8)	
MICU/SICU	5168 (19.1)	4820 (22.6)	348 (6.0)	
Other ICU#	7115 (26.3)	5561 (26.1)	1554 (26.9)	
Year of admission [n (%)]				< 0.001
2008 − 2010	9369 (34.6)	7846 (36.8)	1523 (26.3)	
2011 − 2013	6151 (22.7)	4596 (21.6)	1555 (26.9)	
2014 − 2016	5667 (20.9)	4058 (19.0)	1609 (27.8)	
2017 − 2019	3664 (13.5)	2829 (13.3)	835 (14.4)	
2020 − 2022	2237 (8.3)	1974 (9.3)	263 (4.5)	
LOS before ICU [d, median (IQR)]	1.6 (0.6 − 5.2)	1.5 (0.6 − 3.6)	2.7 (0.5 − 19.1)	< 0.001
Weight [kg, median (IQR)]	78.0 (65.3 − 93.1)	77.4 (64.8 − 92.7)	80.0 (68.0 − 95.0)	< 0.001
**Scores on the first day [score, median (IQR)]**				
GCS	15.0 (13.0 − 15.0)	15.0 (13.0 − 15.0)	15.0 (14.0 − 15.0)	< 0.001
CCI	5.0 (3.0 − 7.0)	5.0 (3.0 − 7.0)	5.0 (3.0 − 6.0)	< 0.001
SOFA	5.0 (3.0 − 8.0)	5.0 (3.0 − 7.0)	7.0 (4.0 − 9.0)	< 0.001
APSIII	46.0 (35.0 − 61.0)	46.0 (35.0 − 60.0)	47.0 (33.0 − 67.0)	< 0.001
**Vital signs and laboratory tests on the first day [median (IQR)]**				
Temperature (℃)	36.9 (36.6 − 37.2)	36.9 (36.6 − 37.2)	36.8 (36.5 − 37.1)	< 0.001
Respiration rate (beat/min)	19.0 (17.0 − 22.0)	19.0 (17.0 − 22.0)	18.0 (16.0 − 21.0)	< 0.001
Heart rate (beat/min)	85.0 (75.0 − 97.0)	85.0 (75.0 − 98.0)	85.0 (77.0 − 97.0)	0. 012
Mean blood pressure (mmHg)	75.0 (70.0 − 82.0)	76.0 (70.0 − 83.0)	74.0 (69.0 − 78.0)	< 0.001
SpO_2 (%)	97.3 (95.8 − 98.5)	97.1 (95.6 − 98.4)	97.8 (96.5 − 98.9)	< 0.001
White blood cell (10^9/L)	11.3 (8.0 − 15.4)	11.2 (7.9 − 15.4)	11.5 (8.5 − 15.2)	0.001
Lymphocyte count (10^9/L)	1.0 (0.6 − 1.6)	1.0 (0.6 − 1.5)	1.2 (0.7 − 1.9)	< 0.001
Neutrophil count (10^9/L)	9.4 (6.0 − 13.7)	9.4 (6.0 − 13.8)	9.3 (6.0 − 13.2)	0.062
Monocyte count (10^9/L)	0.5 (0.3 − 0.9)	0.6 (0.3 − 0.9)	0.5 (0.3 − 0.8)	< 0.001
Platelet (10^9/L)	200.0 (141.0, 271.0)	206.0 (147.0 − 279.0)	174.0 (122.0 − 238.0)	< 0.001

**Variables**	**Total (*n* = 27,088)**	**No albumin (*n* = 21,303)**	**Albumin (*n* =5785)**	***P*-value**
Red cell distribution width (%)	14.6 (13.5 − 16.2)	14.6 (13.5 − 16.2)	14.4 (13.3 − 16.1)	< 0.001
Hemoglobin level (g/dl)	10.3 (8.9 − 11.9)	10.5 (9.0, 12.0)	9.8 (8.4 − 11.3)	< 0.001
Creatinine (mg/dl)	1.0 (0.7 − 1.6)	1.0 (0.7 − 1.6)	0.9 (0.7 − 1.4)	< 0.001
BUN (mg/dl)	20.0 (14.0, 34.0)	21.0 (14.0 − 36.0)	18.0 (14.0 − 29.0)	< 0.001
**Comorbidities [*n* (%)]**				
Cerebrovascular disease				< 0.001
Yes	3879 (14.3)	3278 (15.4)	601 (10.4)	
No	23,209 (85.7)	18,025 (84.6)	5184 (89.6)	
Myocardial infarct				0.837
Yes	4761 (17.6)	3750 (17.6)	1011 (17.5)	
No	22,327 (82.4)	17,553 (82.4)	4774 (82.5)	
Congestive heart failure				< 0.001
Yes	8597 (31.7)	7110 (33.4)	1487 (25.7)	
No	18,491 (68.3)	14,193 (66.6)	4298 (74.3)	
Chronic pulmonary disease				< 0.001
Yes	7445 (27.5)	6025 (28.3)	1420 (24.5)	
No	19,643 (72.5)	15,278 (71.7)	4365 (75.5)	
Renal disease				< 0.001
Yes	6525 (24.1)	5445 (25.6)	1080 (18.7)	
No	20,563 (75.9)	15,858 (74.4)	4705 (81.3)	
Malignant cancer				< 0.001
Yes	3570 (13.2)	2996 (14.1)	574 (9.9)	
No	23,518 (86.8)	18,307 (85.9)	5211 (90.1)	
Liver disease				< 0.001
Yes	4246 (15.7)	2826 (13.3)	1420 (24.5)	
No	22,842 (84.3)	18,477 (86.7)	4365 (75.5)	
Hypertension				< 0.001
Yes	10,757 (39.7)	8092 (38.0)	2665 (46.1)	
No	16,331 (60.3)	13,211 (62.0)	3120 (53.9)	
Diabetes				< 0.001
Yes	8697 (32.1)	7003 (32.9)	1694 (29.3)	
No	18,391 (67.9)	14,300 (67.1)	4091 (70.7)	
Septic shock				0.579
Yes	5608 (20.7)	4426 (20.8)	1182 (20.4)	
No	21,480 (79.3)	16,877 (79.2)	4603 (79.6)	
Chronic kidney disease				< 0.001
Yes	6395 (23.6)	5343 (25.1)	1052 (18.2)	
No	20,693 (76.4)	15,960 (74.9)	4733 (81.8)	
**Drugs and treatments on the first day**				
Time to antibiotic use [h, median (IQR)]	7.4 (2.7 − 16.0)	7.7 (3.5 – 16.0)	5.8 (0.2 − 16.4)	< 0.001
Total amount of crystalloids dosage [ml, median (IQR)]	2480.0 (1290.8 − 4000.0)	2293.0 (1140.5 − 3759.5)	3205.0 (1965.0 − 4813.0)	< 0.001

Variables	Total (*n* = 27,088)	No albumin (*n* = 21,303)	Albumin (*n* =5785)	*P*-value
Artificial colloid [*n* (%)]				1.000
Yes	19 (0.1)	15 (0.1)	4 (0.1)	
No	27,069 (99.9)	21,288 (99.9)	5781 (99.9)	
Nephrotoxic drug use$ [*n* (%)]				< 0.001
Yes	9445 (34.9)	7191 (33.8)	2254 (39.0)	
No	17,643 (65.1)	14,112 (66.2)	3531 (61.0)	
Mechanical ventilation [*n* (%)]				< 0.001
Yes	12,326 (45.5)	8337 (39.1)	3989 (69.0)	
No	14,762 (54.5)	12,966 (60.9)	1796 (31.0)	
Vasoactive agent [*n* (%)]				< 0.001
Yes	11,545 (42.6)	7335 (34.4)	4210 (72.8)	
No	15,543 (57.4)	13,968 (65.6)	1575 (27.2)	
**Outcomes**				
SA-AKI [*n* (%)]				< 0.001
Yes	21,017 (77.6)	15,955 (74.9)	5062 (87.5)	
No	6071 (22.4)	5348 (25.1)	723 (87.5)	
7-day mortality [*n* (%)]				< 0.001
Expired	2356 (8.7)	1949 (9.1)	407 (7.0)	
Alive	24,732 (91.3)	19,354 (90.9)	5378 (93.0)	

*Other includes individuals identified as Asian, Black, Hispanic/Latino, and Other. #Other ICU includes the following units with smaller sample sizes or mixed clinical profiles: intensive care unit, med/surg intensive care unit, medicine intensive care unit, medicine/cardiology intermediate, neuro intermediate, neuro stepdown, neuro surgical intensive care unit, post anesthesia care unit, surgery/trauma intensive care unit, surgery/vascular/intermediate, and trauma surgical intensive care unit. $The use of nephrotoxic drugs refers to administration from hospital admission until the occurrence of SA-AKI, including vancomycin, gentamicin, amikacin, tobramycin, colistin, polytrim and amphotericin [8]. *BUN* blood urea nitrogen, *ICU* intensive care unit, *CCU* coronary care unit, *CVICU* cardiovascular intensive care unit, *MICU* medical intensive care unit, *SICU* surgical intensive care unit, *LOS* length of stay, *GCS* glasgow coma scale, *CCI* charlson comorbidity index, *SOFA* sequential organ failure assessment, *APSIII* acute physiology score III, SpO_2 peripheral capillary oxygen saturation

Table S6 Sensitivity analysis: outcomes in patients with sepsis in the ICU after clone-censor-weight (follow-up grace period of 12 h)

**Outcomes (*n* = 54,176)**	**No albumin group (*n* = 27,088)**	**Albumin group (*n* = 27,088)**
**Primary outcome**		
SA-AKI (%)	90.97	94.71
Difference in RMTL [h, % (95% CI*)]	9.40 (7.04 − 11.78)	
Difference in SA-AKI risk [% (95% CI*)]	3.74 (1.66 − 5.92)	
**Secondary outcome**		
7-day survival (%)	85.53	85.54
Difference in RMST [h, % (95% CI*)]	0.38 (-1.33 to 2.14)	
Difference in 7-day survival [% (95% CI*)]	0.01 (-2.84 to 2.97)	

*All 95% CIs were calculated from 1000 bootstrap replicates. *ICU* intensive care unit, *SA-AKI* sepsis-associated acute kidney injury, *RMTL* restricted mean time lost, *CI* confidence interval, *RMST* restricted mean survival time

Table S7 Sensitivity analysis: outcomes in patients with sepsis in the ICU after clone-censor-weight (follow-up grace period of 36 h)

**Outcomes (*n* = 54,176)**	**No albumin group (*n* = 27,088)**	**Albumin group (*n* = 27,088)**
**Primary outcome**		
SA-AKI (%)	90.57	93.42
Difference in RMTL [h, % (95% CI*)]	6.04 (4.38 − 7.67)	
Difference in SA-AKI risk [% (95% CI*)]	2.85 (1.07 − 4.65)	
**Secondary outcome**		
7-day survival (%)	85.16	86.02
Difference in RMST [h, % (95% CI*)]	1.34 (0.19 − 2.50)	
Difference in 7-day survival [% (95% CI*)]	0.86 (-1.25 to 2.97)	

*All 95% CIs were calculated from 1000 bootstrap replicates. *ICU* intensive care unit, *SA-AKI* sepsis-associated acute kidney injury, *RMTL* restricted mean time lost, *CI* confidence interval, *RMST* restricted mean survival time

Table S8 Sensitivity analysis: outcomes in patients with sepsis in the ICU after clone-censor-weight (excluding those who received RRT in the primary and secondary analysis)

**Outcomes**	**No albumin group**	**Albumin group**
**Primary outcome (*n* = 26,737)**	*n* = 23,575	*n* = 3162
SA-AKI (%)	90.65	94.20
Difference in RMTL [h, % (95% CI*)]	7.79 (5.87 − 9.56)	
Difference in SA-AKI risk [% (95% CI*)]	3.55 (1.62 − 5.33)	
**Secondary outcome (*n* = 25,083)**	*n* = 20,018	*n* = 5065
7-day survival (%)	86.14	87.56
Difference in RMST [h, % (95% CI*)]	1.65 (0.19 − 3.01)	
Difference in 7-day survival [% (95% CI*)]	1.42 (-1.04 to 3.84)	

*All 95% CIs were calculated from 1000 bootstrap replicates. *ICU* intensive care unit, *SA-AKI* sepsis-associated acute kidney injury, *RMTL* restricted mean time lost, *CI* confidence interval, *RMST* restricted mean survival time

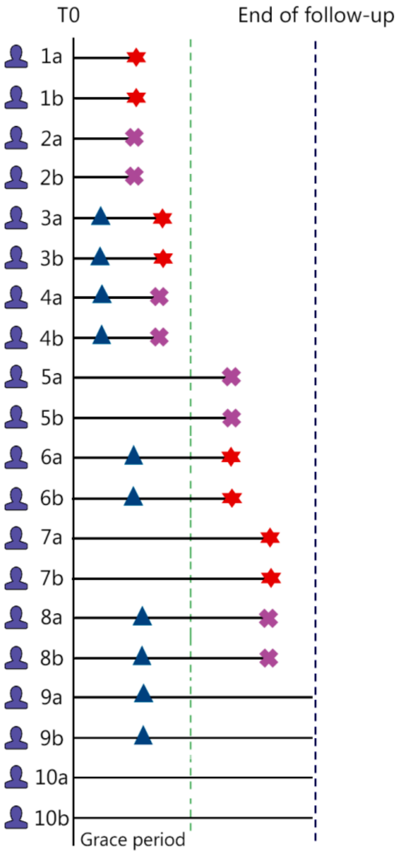

Clone	Albumin	Censor	Outcome
0	0	0	1
1	0	0	1
0	0	0	0
1	0	0	0
0	1	1	0
1	1	0	1
0	1	1	0
1	1	0	0
0	0	0	0
1	0	1	0
0	1	1	0
1	1	0	1
0	0	0	1
1	0	1	0
0	1	1	0
1	1	0	0
0	1	1	0
1	1	0	0
0	0	0	0
1	0	1	0

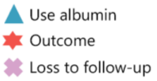

Fig. S1 The clone-censor mechanism in this study

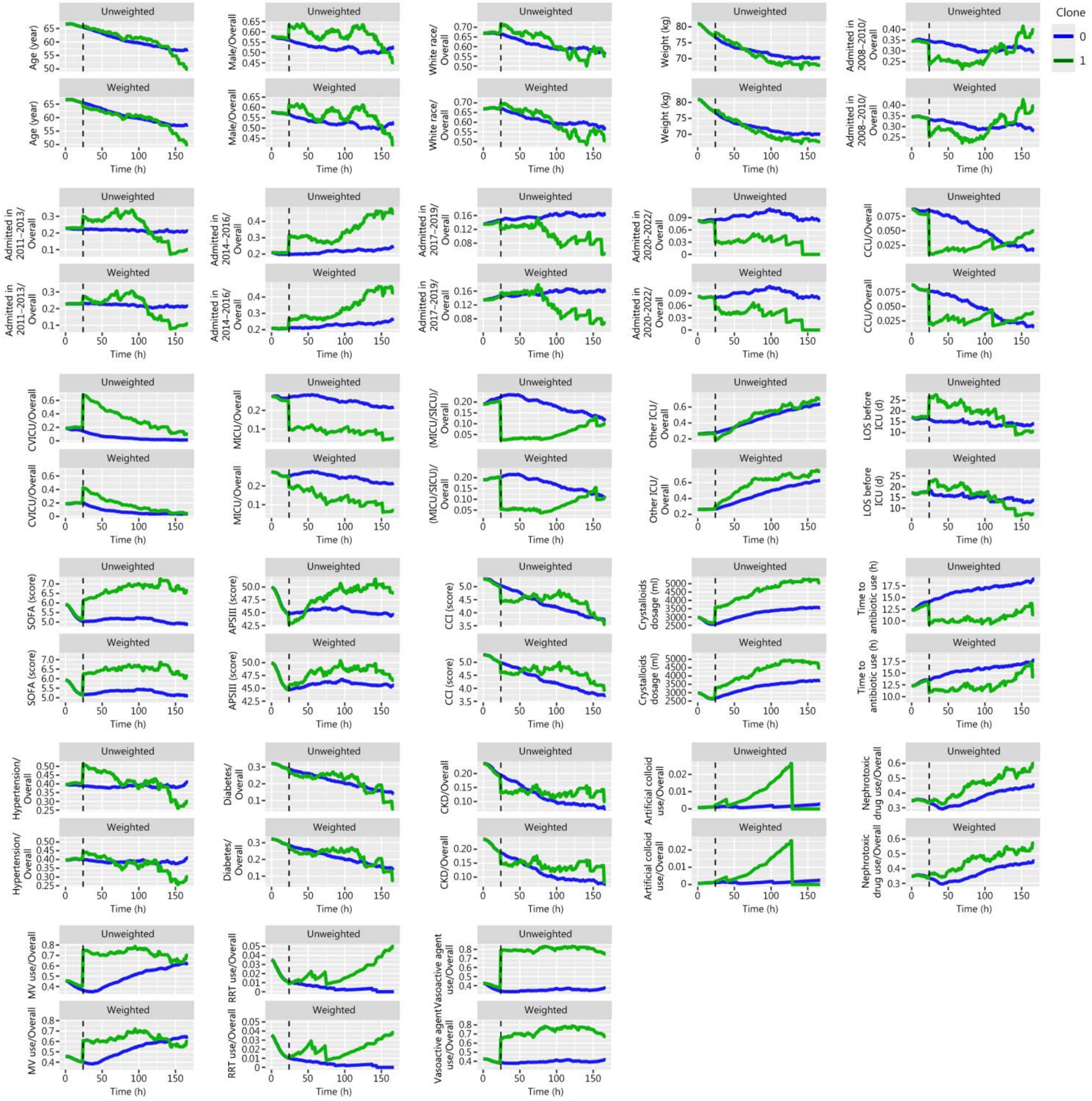

Fig. S2 Average of changes over time before and after weighting of covariates in the primary analysis. LOS length of stay, ICU intensive care unit, CCU coronary care unit, CVICU cardiovascular intensive care unit, MICU medical intensive care unit, SICU surgical intensive care unit, SOFA sequential organ failure assessment, APSIII acute physiology score III, CCI charlson comorbidity index, CKD chronic kidney disease, MV mechanical ventilation, RRT renal replacement therapy

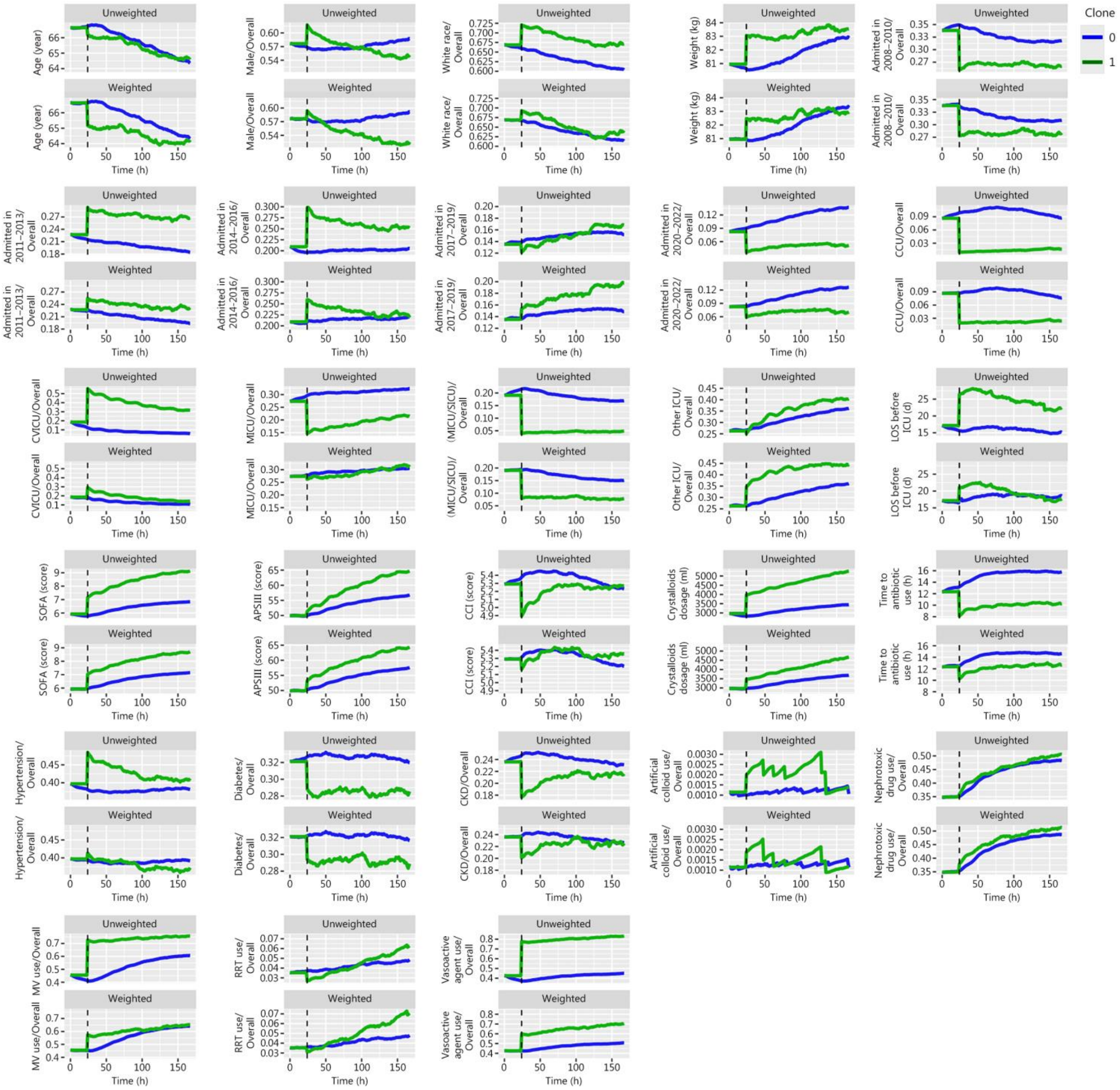

Fig. S3 Average of changes over time before and after weighting of covariates in the secondary analysis. LOS length of stay, ICU intensive care unit, CCU coronary care unit, CVICU cardiovascular intensive care unit, MICU medical intensive care unit, SICU surgical intensive care unit, SOFA sequential organ failure assessment, APSIII acute physiology score III, CCI charlson comorbidity index, CKD chronic kidney disease, MV mechanical ventilation, RRT renal replacement therapy

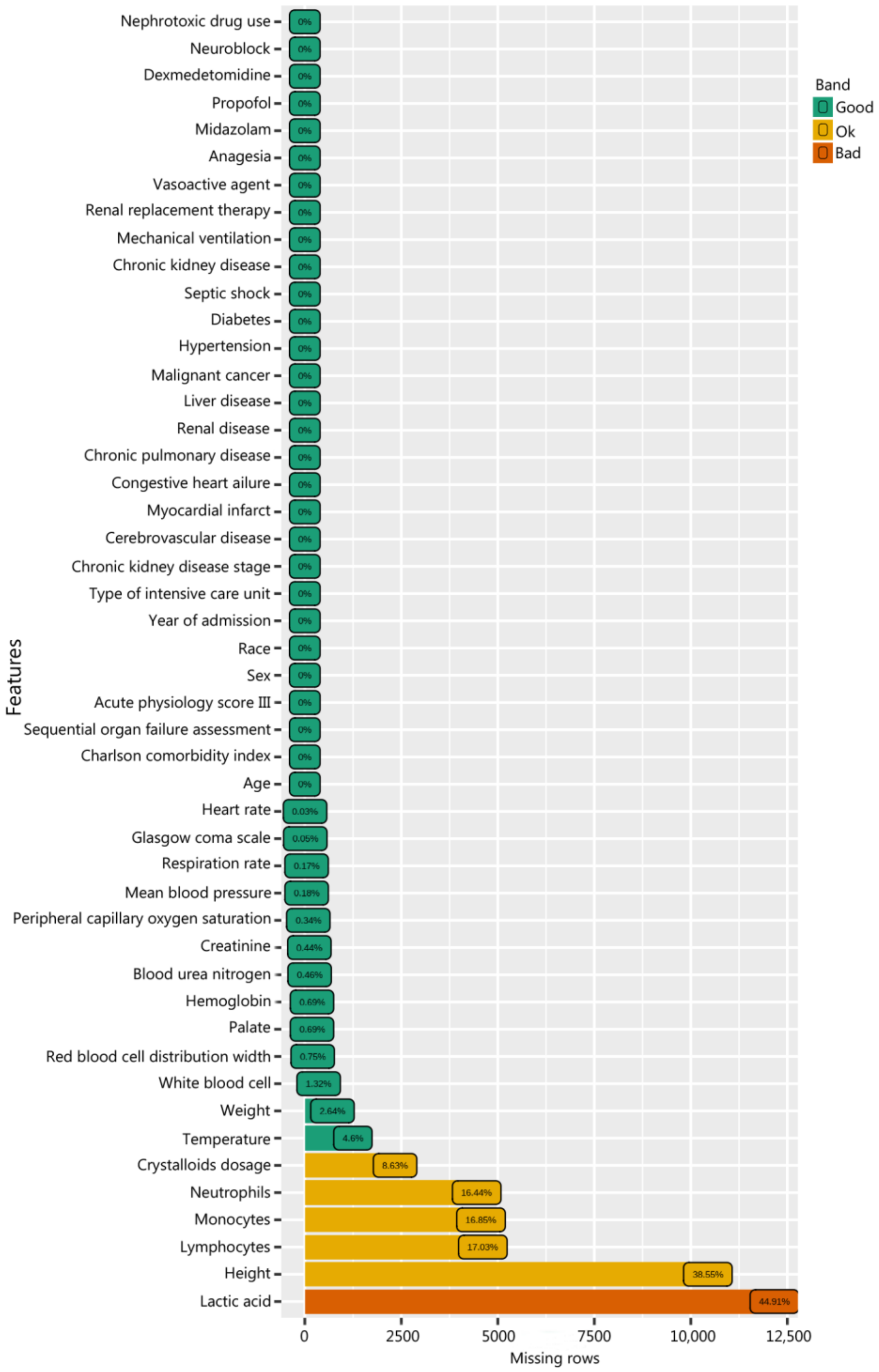

Fig. S4 Missing data for all variables in this study

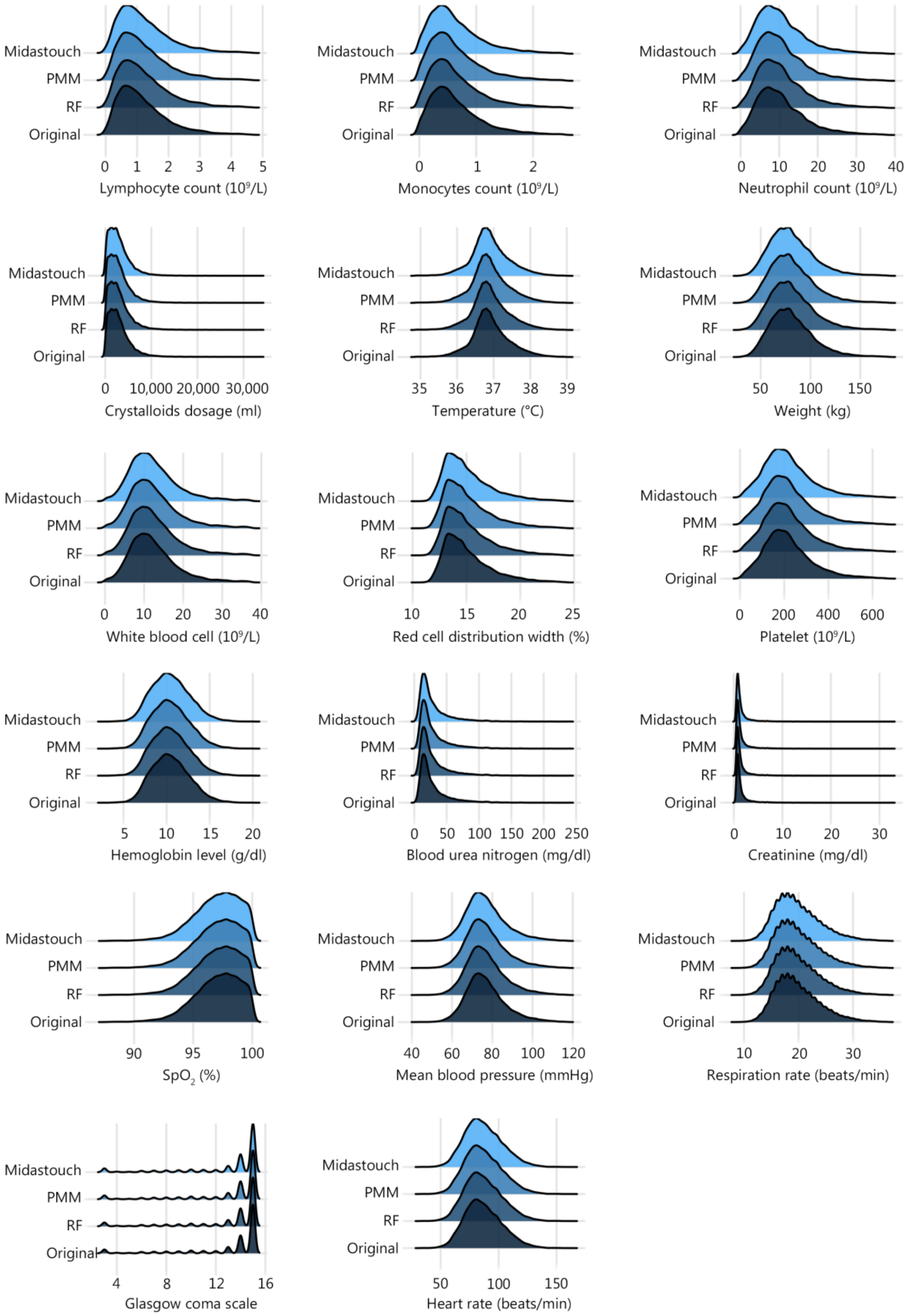

Fig. S5 Comparison of multiple imputation of candidate features. PMM predictive mean matching, RF random forest, SpO_2 peripheral capillary oxygen saturation

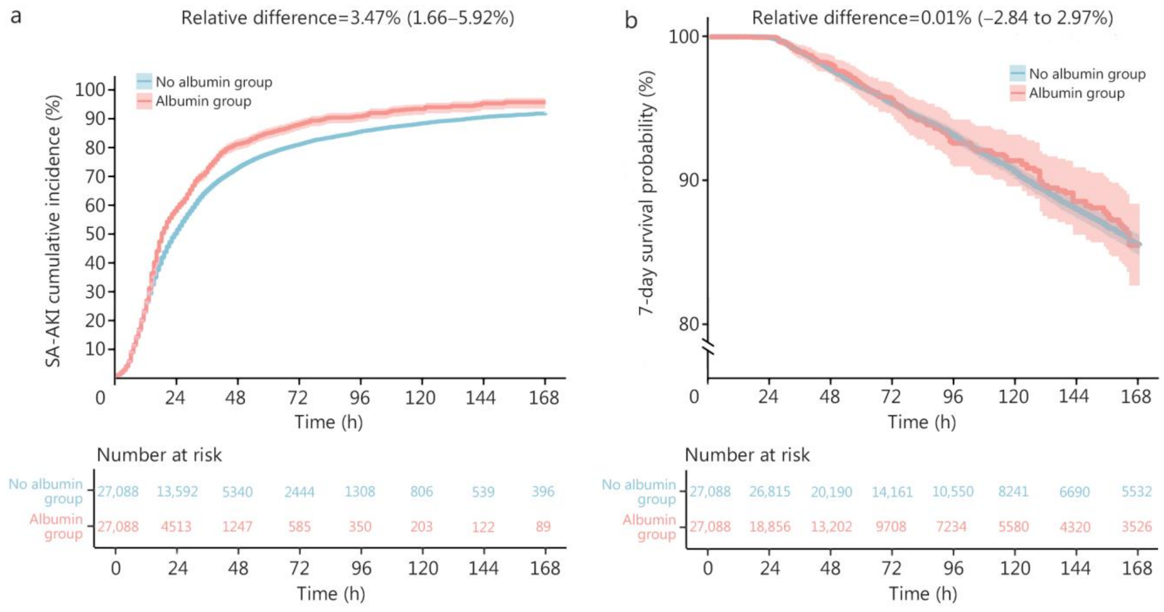

Fig. S6 SA-AKI cumulative risk curve and 7-day Kaplan-Meier survival curve (follow-up grace period of 12 h). **a** SA-AKI cumulative risk curve. **b** 7-day Kaplan-Meier survival curve. All 95% CIs were calculated from 1000 bootstrap replicates. SA-AKI sepsis-associated acute kidney injury, CI confidence interval

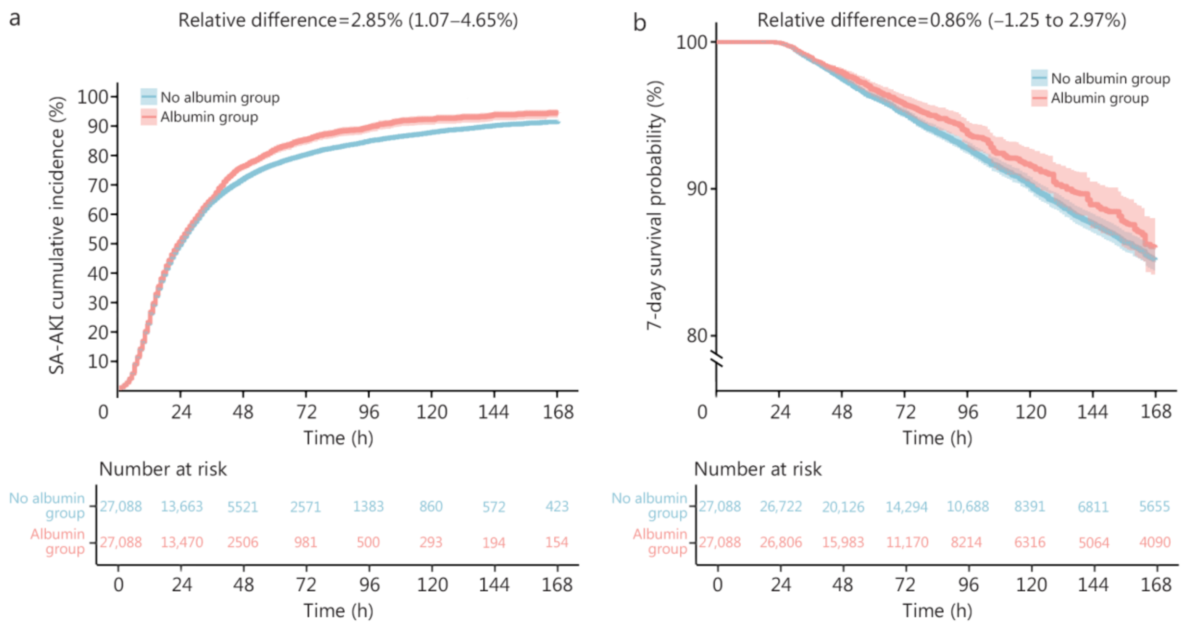

Fig. S7 SA-AKI cumulative risk curve and 7-day Kaplan-Meier survival curve (follow-up grace period of 36 h). **a** SA-AKI cumulative risk curve. **b** 7-day Kaplan-Meier survival curve. All 95% CIs were calculated from 1000 bootstrap replicates. SA-AKI sepsis-associated acute kidney injury, CI confidence interval

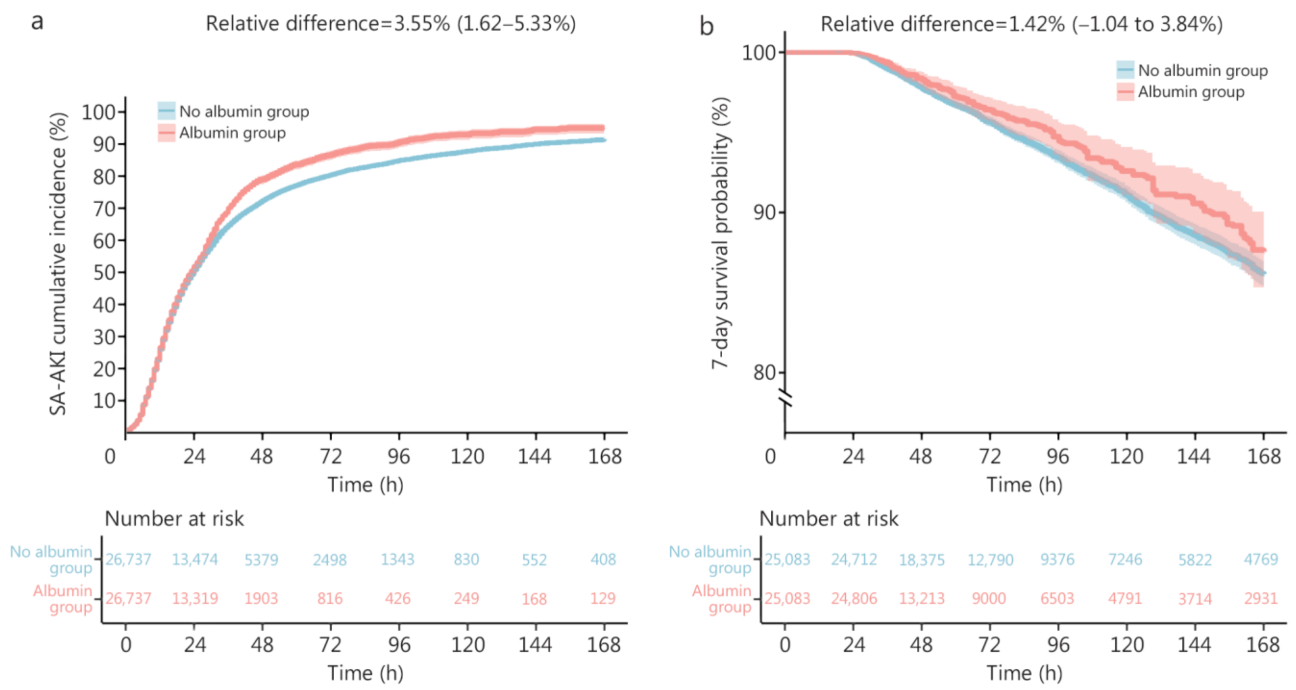

Fig. S8 SA-AKI cumulative risk curve and 7-day Kaplan-Meier survival curve (excluding those who received RRT). **a** SA-AKI cumulative risk curve. **b** 7-day Kaplan-Meier survival curve. All 95% CIs were calculated from 1000 bootstrap replicates. SA-AKI sepsis-associated acute kidney injury, CI confidence interval

Reference

1. Gaber CE, Hanson KA, Kim S, Lund JL, Lee TA, Murray EJ. The clone-censor-weight method in pharmacoepidemiologic research: foundations and methodological implementation. Curr Epidemiol Rep. 2024: 11, 164-74.
2. Wong CKH, Lau KTK, Chung MSH, Au ICH, Cheung KW, Lau EHY, et al. Nirmatrelvir/ritonavir use in pregnant women with SARS-CoV-2 Omicron infection: a target trial emulation. Nat Med. 2024;30(1):112-6.
3. Gaber CE, Ghazarian AA, Strassle PD, Ribeiro TB, Salas M, Maringe C, et al. De-mystifying the clone-censor-weight method for causal research using observational data: a primer for cancer researchers. Cancer Med. 2024;13(23):e70461.
4. Wang GH, Lai EC, Goodin AJ, Reise RC, Shorr RI, Lo-Ciganic WH. Injurious fall risk differences among older adults with first-line depression treatments. JAMA Netw Open. 2024;7(8):e2435535.
5. Maringe C, Benitez Majano S, Exarchakou A, Smith M, Rachet B, Belot A, Leyrat C. Reflection on modern methods: trial emulation in the presence of immortal-time bias. Assessing the benefit of major surgery for elderly lung cancer patients using observational data. Int J Epidemiol. 2020;49(5):1719-29.
6. Hernán MA. How to estimate the effect of treatment duration on survival outcomes using observational data. BMJ. 2018;360:k182.
7. Hernán MA, Robins JM. Using big data to emulate a target trial when a randomized trial is not available. Am J Epidemiol. 2016;183(8):758-64.
8. Petejova N, Martinek A, Zadrazil J, Kanova M, Klementa V, Sigutova R, et al. Acute kidney injury in septic patients treated by selected nephrotoxic antibiotic agents-pathophysiology and biomarkers-a review. Int J Mol Sci. 2020;21(19):7115.
